# Faster disease progression in Parkinson's disease with glucocerebrosidase genotype: But not apparent immediate from diagnosis

**DOI:** 10.1177/1877718X251361507

**Published:** 2025-09-03

**Authors:** Henrieke L Frequin, Bart Ferwerda, Constant VM Verschuur, Sven R Suwijn, Joke M Dijk, Rob MA de Bie, for the LEAP Study Group

**Affiliations:** 1 Department of Neurology, Amsterdam University Medical Center (Amsterdam UMC), Amsterdam Neuroscience, University of Amsterdam, Amsterdam, The Netherlands; 2 Department of Clinical Epidemiology and Biostatistics, Amsterdam University Medical Center (Amsterdam UMC), University of Amsterdam, Amsterdam, The Netherlands; 3 Department of Neurology, Albert Schweitzer ziekenhuis, Dordrecht, the Netherlands

**Keywords:** Parkinson's disease, glucocerebrosidase, genotype, phenotype, disease progression

## Abstract

**Background:**

This study presents post-hoc analyses of the LEAP study focusing on disease progression in patients with early Parkinson's disease (PD) who either have a glucocerebrosidase gene (*GBA1*) mutation (GBA1mut) or do not have a mutation (GBA1wt) over a period of up to five years.

**Objective:**

To investigate the difference in disease progression between GBA1mut and GBA1wt over 80 weeks and five years.

**Methods:**

The study analyzed the difference in disease progression between GBA1mut and GBA1wt using the UPDRS and its subscales, Levy A and B scores, and the difference in levodopa equivalent daily dose (LEDD) over 80 weeks and five years, with mixed-effects regression models.

**Results:**

The *GBA1* mutation carrier status was determined in 394 patients, with 52 being GBA1mut and 342 being GBA1wt. From baseline to 80 weeks, the change in total UPDRS score was similar for GBA1mut and GBA1wt (difference 1.7 points in favor of GBA1mut, p = 0.38). From baseline to five years, GBA1mut had 5.9 points (p = 0.04) more worsening of total UPDRS compared to GBA1wt and GBA1mut had 1.0 point (p = 0.02) more deterioration in UPDRS subscale IV, related to therapy complications, compared to GBA1wt. There were no significant between-group differences in changes in UPDRS subscales, Levy A and B scores, and LEDD.

**Conclusions:**

These findings suggest that over the long term, PD patients with a *GBA1* mutation experience faster disease progression compared to those without a *GBA1* mutation, although this difference in progression was not apparent within the initial 80 weeks of the trial.

## Plain language summary

Parkinson's disease (PD) is a multifactorial disease. DNA mutations in the glucocerebrosidase (*GBA*1) gene are the most common genetic risk factor for PD (10% of patients with European descent). At the moment of diagnosis patients with a *GBA*1 mutation are slightly younger but have the same symptom severity as patients without a *GBA*1 mutation. But later in the disease course, however not clear from when exactly, their symptoms start to progress faster. A study concerning disease progression in early PD patients during five years had the *GBA*1 mutation carrier status successfully determined in 394 patients. This gave us the opportunity to study disease progression in patients with a *GBA*1 mutation compared to patients without a *GBA*1 mutation. These 394 patients were examined for symptom severity, amount of antiparkinson medication and side effects of this medication 8 times during the first 80 weeks of the study and five years after start of the study. A total of 52 patients had a *GBA*1 mutation. We found more deterioration of symptoms and more side effects of antiparkinson medication in patients with a *GBA*1 mutation after five years, but not yet during the first 80 weeks. This information could help physicians counseling PD patients with a *GBA*1 mutation and it could help researchers studying *GBA*1 targeted therapies.

## Introduction

The glucocerebrosidase gene (*GBA1*) encodes for the lysosomal enzyme β-glucocerebrosidase. Mutations in this gene are the most common genetic risk factor for Parkinson's disease (PD).^1–3^ PD patients with a *GBA1* mutation (GBA1mut) have a younger age at disease onset, a faster decline of motor, non-motor and cognitive functions, and have a shortened life expectancy compared to PD patients without a *GBA1* mutation (GBA1wt).^1,3–14^ Mutations in the *GBA1* gene are classified as ‘severe’, ‘mild’, or ‘polymorphisms/common variants’ based on their association with Gaucher's Disease^15^ The risk of PD is higher in individuals with severe mutations compared to those with mild mutations or polymorphisms. Severe *GBA1* mutations are also linked to an increased risk of developing dementia.^9,13^ However, clear differences in the progression of motor symptoms between severe and mild mutations, or polymorphisms, have not yet been demonstrated.^9,13^

Findings concerning disease progression in GBA1mut patients are mostly based on retrospective data, on prospectively gathered data from PD patients with advanced disease,^1,4–9,11–14^ and on fewer data from incidence cohorts in which clinical assessments were performed with intervals of six, 12 or 18 months over a longer time course.^3,10^ GBA1mut patients are clinically indistinguishable from GBA1wt patients at the moment of PD diagnosis.^3,10^ It is unclear when in the course of the disease symptoms progress faster in GBA1mut patients. This information would be relevant for counseling patients, but also for trial design of disease modifying treatments as these treatments are often studied in patients with early PD over the time course of about two years.^16^

We performed post-hoc analyses of the Levodopa in EArly Parkinson's disease (LEAP) study concerning the association between *GBA1* mutation carrier status and disease progression in early PD patients.^17^ Eight study visits were performed within the first 80 weeks of the trial, with prospective follow-up visits three and five years after baseline.^17^

## Methods

### LEAP study design

The LEAP study was a randomized, placebo-controlled, delayed-start trial in which patients were randomly assigned to treatment with levodopa/carbidopa 300/75 mg per day for 80 weeks (i.e., early-start group) or to treatment with placebo for 40 weeks followed by levodopa/carbidopa 300/75 mg per day for 40 weeks (i.e., delayed-start group). Detailed methods of the study were published previously.^17,18^ Study visits were performed at baseline before start of study medication and at weeks 4, 22, 40, 44, 56, 68, and 80. After 80 weeks, treatment was no longer dictated by a study protocol. The prospective follow-up comprised study visits three and five years after baseline. Due to a delay in funding, the three-year follow-up visits started after some of the patients had already passed the three-year follow-up time frame. Every visit we recorded the Unified Parkinson's disease Rating Scale (UPDRS) score and the amount of antiparkinson medication from which we calculated the levodopa equivalent daily dose (LEDD).^19,20^ From the UPDRS score, we derived the Levy A and Levy B scores. Levy A refers to the sum of UPDRS part III items considered levodopa-response: facial expression, tremor, rigidity, hand movements, pronation-supination movements of hands, leg agility and global spontaneity of movement (range: 0–80 points).^15^ Levy B refers to the sum of UPDRS part III items associated with axial features of the disease, which are considered less responsive to levodopa: speech, arising from chair, posture, gait and postural stability (range: 0–20 points).^21^ At baseline, the Mini-Mental State Examination (MMSE) was obtained.^22^ At five years, the Scales for Outcomes in Parkinson's disease-Autonomic (SCOPA-AUT) and the Montreal Cognitive Assessment (MoCA) were administered.^23,24^ The SCOPA-AUT score ranges from 0 to 69, with higher scores indicating more severe autonomic dysfunction. The MMSE and MoCA score range from 0 to 30, with lower scores indicating more severe cognitive dysfunction. We inquired after the diagnosis (i.e., PD or another diagnosis) of all included patients with their treating neurologist five years after baseline. The treating neurologists were either general neurologists or movement disorder specialists.

### Patients

Patients were recruited from academic and community hospitals in the Netherlands between August 2011 and May 2016. Patients were eligible for enrolment if they had received a diagnosis of PD within the previous two years from an experienced neurologist,^17,18^ if they had insufficient disability to warrant treatment with antiparkinson medication, if they were 30 years of age or older, and if they had a life expectancy of more than two years. Patients who had been treated with antiparkinson medication previously were excluded. Patients were also excluded if their most prominent symptom was tremor, such as a severe resting tremor that was present almost continuously or resulted in disability; if they had dementia; and if they had features that indicated atypical or secondary parkinsonism.^17,18^

### Standard protocol approvals, registrations and patient consents

The protocol was approved by the ethics committee at the Amsterdam University Medical Center in the Netherlands. The trial was conducted in accordance with the principles of the Declaration of Helsinki. Trial monitoring and data management were performed in accordance with the International Conference on Harmonisation Good Clinical Practice guidelines. All the patients provided written informed consent. The LEAP study was registered with the ISRCTN registry, number ISRCTN30518857, and at the European Union Drug Regulating Authorities Clinical Trials Database (EudraCT), number 2011-000678-72.^17,18^

### Genetic analysis

Peripheral blood samples were taken at baseline and genomic DNA was extracted with the Gentra Puregene Isolation Kit (Qiagen). *GBA1* missense mutations were determined by sequencing the complete *GBA1* gene using the Illumina NovaSeq 6000 Next generation sequencing platform. Paired-End 150 bp sequencing of the human *GBA1* gene was performed at GenomeScan (GenomeScan BV, Leiden, NL). Quality control, alignment, recalibration and variant calling was performed by GenomeScan. In summary, mapping was performed with BWA v0.7.4, using the hg19 reference genome obtained from the UCSC website.^25^ The *GBAP1* pseudogene was masked during mapping. Variant calling was performed using the GATK VariantCall v3.^26^ Patients were screened for the presence of *GBA1* missense mutations including *GBA1* polymorphisms. All found missense mutations were manually inspected and reviewed within the aligned BAM files using Integrative Genomics Viewer.^27^

### Statistical analysis

All further analyses only included patients who still had the diagnosis PD five years after baseline and in whom the *GBA1* mutation carrier status was successfully determined. We compared baseline clinical characteristics of patients with a mild or severe *GBA1* mutation with patients without a mutation (i.e., GBA1wt), and of patients with a GBA1 polymorphism with GBA1wt. We analyzed the association between GBA1 mutation carrier status (i.e., GBA1 mutation and polymorphism together—abbreviated as GBA1mut—versus GBA1wt) and disease progression measured by total UPDRS (i.e., sum of UPDRS parts I, II and III), UPDRS sub scores, and Levy A and Levy B scores from baseline to 80 weeks and from baseline to five years using a mixed-effects regression model using the R package lme4. We performed the same analyses on the progression of the total UPDRS score and UPDRS subscores from baseline to five years for patients with *GBA1* polymorphisms versus GBA1wt and for patients with *GBA1* mutations (mild and severe grouped together) versus GBA1wt. Analyses were performed with adjustment for age, gender, disease duration, and LEDD.^20^ In the fixed part of the model, disease progression was modelled as a linear function of time and LEDD. In the random part of the model, both intercept and slope of the regression lines of UPDRS score or Levy score on time were allowed to vary between patients. Missing data was omitted pairwise. We analyzed the association between *GBA1* mutation carrier status and cognitive dysfunction at five years by use of the MoCA score and between *GBA1* mutation carrier status and autonomic dysfunction at five years with the SCOPA-AUT, both using regression models. These analyses are performed with adjustment for LEDD, age, disease duration, and gender. We analyzed the association between change in LEDD and *GBA1* mutation carrier status over 80 weeks and over five years by use of a regression model with adjustment for age, disease duration, and gender. We analyzed the difference between GBA1mut versus GBA1wt in prevalence of hallucinations (UPDRS item 2), motor response fluctuations (UPDRS item 39) and dyskinesia (UPDRS item 32) at 80 weeks and at five years with chi-squared tests.

Two-tailed p-values less than 0.05 were considered statistically significant. We did not apply corrections for multiple comparisons, as the analyses were exploratory in nature, aimed at investigating the potential impact of GBA1 mutation carrier status on various aspects of disease progression.^28^

## Results

Of the 445 patients randomized in the LEAP study, 27 patients were excluded because five years after baseline it was found that they did not have PD, and 24 patients were excluded because they did not consent to analysis of their *GBA1* carrier status or because their blood sample did not pass quality control. In 52 patients (13%), we found a missense mutation in the *GBA1* gene. A total of 44 patients had a *GBA1* polymorphism (i.e., rs2230288/E326K or rs75548401/T369M) and eight patients had a mild or severe GBA1 mutation (i.e., rs76763715/N370S or respectively rs421016/L444P). Table 1 provides further specifications. The baseline characteristics of the included patients did not differ significantly per *GBA1* mutation carrier status group (Table 2). There were no significant differences in baseline characteristics between the patients with a known versus an unknown *GBA1* mutation carrier status (Table 2).

**Table 1.** GBA1 missense mutations*.

	Any missense mutation	Polymorphisms			Mild and severe mutations		
		E326K	D140H + E326K	T369M	L444P	L444P + A456P	N370S
All patients, N	52	26	7	11	4	2	2

*Mutations in the *GBA*1 gene are defined as ‘severe’, ‘mild’ or as ‘polymorphisms’, based on their association with Gaucher's Disease.

**Table 2.** Baseline characteristics.

	GBA1wt	*GBA1* polymorphism	p*	*GBA1* mild or severe mutation	p**	*GBA1* mutation carrier status unknown	p***
Total, N	342	44		8		24	
Male, N (%)	245 (72)	26 (59)	0.11	5 (63)	0.69	16 (67)	0.64
Age start study	68.8 ± 8.8	66.5 ± 8.5	0.42	66.9 ± 7.0	0.49	69.8 ± 7.6	0.67
Age onset symptoms	65.3 ± 8.7	63.3 ± 8.7	0.10	63.9 ± 6.7	0.47	66.1 ± 8.1	0.53
Treatment group early (%)	163 (48)	24 (60)	0.15	5 (63)	0.57	13 (54)	0.64
UPDRS total	28.5 ± 11.6	28.6 ± 10.6	0.99	29.9 ± 11.6	0.76	29.5 ± 14.6	0.77
UPDRS I	2.3 ± 1.3	2.5 ± 1.3	0.35	2.3 ± 1.3	0.84	2.5 ± 1.4	0.71
UPDRS II	7.2 ± 3.4	7.5 ± 3.7	0.57	8.8 ± 3.3	0.23	8.1 ± 4.7	0.38
UPDRS III	19.0 ± 9.1	18.5 ± 8.1	0.72	18.9 ± 9.0	0.97	18.9 ± 10.3	0.97
UPDRS IV	0.6 ± 0.8	0.5 ± 0.8	0.31	1.3 ± 1.2	0.18	0.6 ± 0.8	0.79
Levy A	14.1 ± 7.0	14.0 ± 6.2	0.91	13.1 ± 8.1	0.75	14.5 ± 8.0	0.82
Levy B	2.5 ± 1.9	2.3 ± 1.9	0.45	3.1 ± 2.0	0.40	2.5 ± 1.9	0.88
MMSE (IQR)	29 (28–30)	29 (28–30)	0.62	29 (28–30)	0.67	29 (27–30)	0.40

Age, UPDRS and Levy scores are presented as means ± SD.

Independent samples T-tests, chi-squared tests and Mann Whitney U tests were used for comparisons.

Age onset symptoms refers to the moment the patient recalled to notice the first PD symptoms.

GBA1wt refers to patient without a *GBA1* polymorphism or mild or severe *GBA1* mutation.

Treatment group early refers to the number of patients who were randomized to the early-start group in the LEAP study protocol.

UPDRS total refers to the sum of UPDRS part I, II and III.

Characteristics of patients with a *GBA1* polymorphism (p value*) and a mild or severe *GBA1* mutation (p value**) were both compared to GBA1wt.

Characteristics of patients of whom their *GBA1* mutation carrier status was not known were compared to all patient of whom the *GBA1* carrier status was known (p value***).

**Figure 1.**
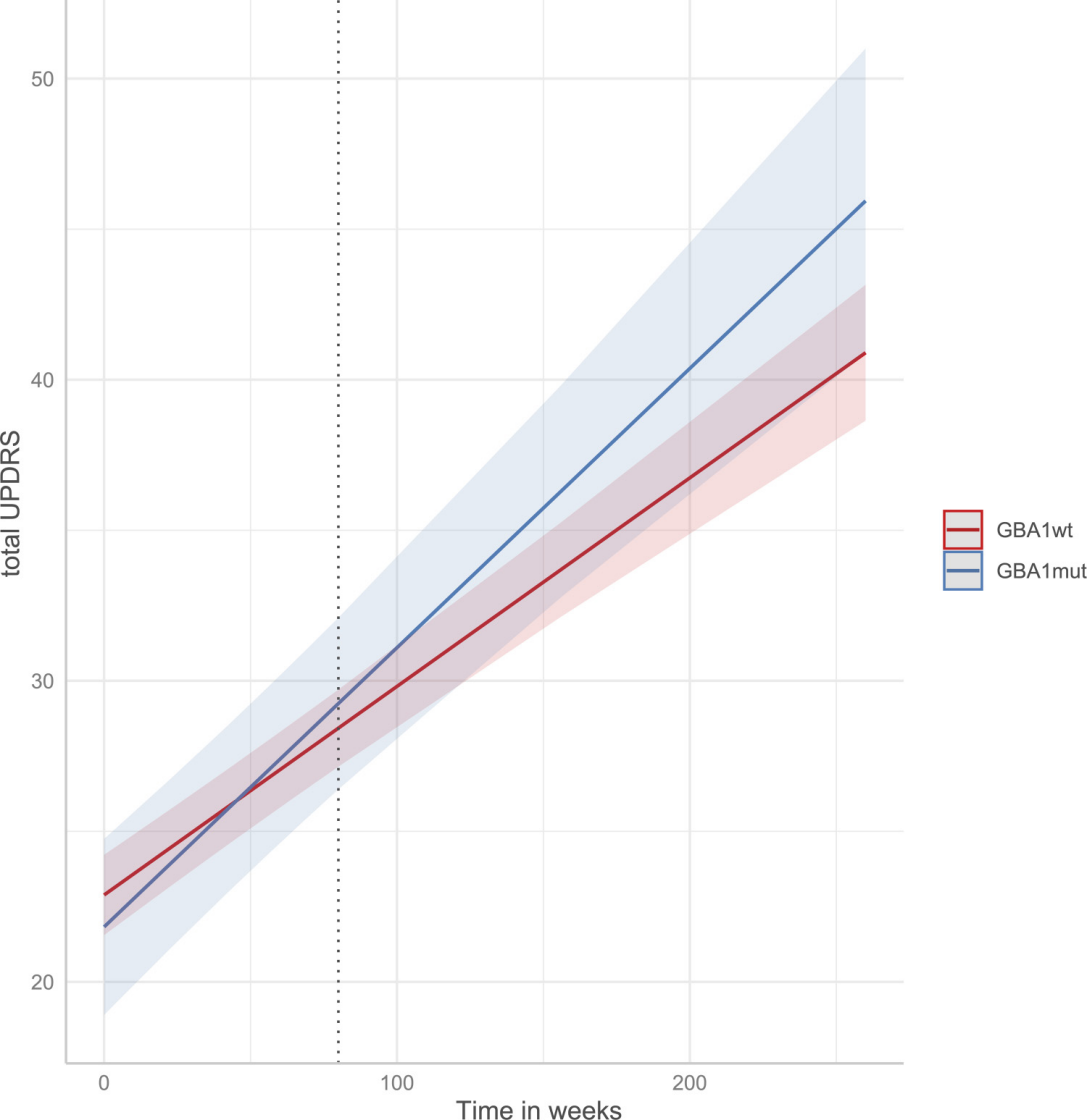
Predicted total UPDRS score for GBA1mut and GBA1wt over the course of five years*. Total UPDRS: sum of UPDRS I, II, and III. GBA1wt: patients without a *GBA1* polymorphism, mild or severe *GBA1* mutation. GBA1mut: patients with a *GBA1* polymorphism, mild or severe *GBA1* mutation. * by mixed-effects regression model.

The mixed-effects regression model with adjustment for age, gender, age of start symptoms, and LEDD showed that patients with any (i.e., polymorphism, mild and severe) GBA1 missense mutation (i.e., GBA1mut) had 1.7 points (95% CI −0.5 to 2.0, p = 0.38) less worsening of total UPDRS score (i.e., sum of UPDRS part I, II and III) over 80 weeks but 5.9 points (0.3 to 11.7, p = 0.04) more worsening of total UPDRS score over five years compared to GBA1wt (Figure 1). The models for the separate UPDRS scores, Levy A and B scores, and LEDD showed no significant differences between GBA1mut and GBA1wt over 80 weeks (Table 3). From baseline to five years, GBA1mut had significantly more worsening of UPDRS subscale IV, concerning complications of therapy (difference 1.0 point, 95% CI 0.2 to 1.8, p = 0.02). There were no significant differences between GBA1mut and GBA1wt over five years in the other UPDRS subscales, Levy A and B scores, and LEDD (Table 3). Although, there were non-significant trends towards more worsening of UPDRS I (0.6 points, 95% CI −0.5 to 1.2, p = 0.07) and UPDRS II (4.1 points, 95% CI 0 to 8.3, p = 0.05) in GBA1mut versus GBA1wt over five years. Patients with a *GBA1* polymorphism tended to deteriorate faster compared to patients with GBA1wt (difference of 5.8 points in the UPDRS total score, p = 0.06). The change in UPDRS total score and subscores from baseline to five years for patients with *GBA1* polymorphisms and those with *GBA1* mild or severe mutations are provided in the Supplemental Material.

**Table 3.** Outcomes mixed-effects regression model, change from baseline to 80 weeks and from baseline to five years.

	Change from baseline to 80 weeks				Change from baseline to five years			
	GBA1wt	GBA1mut	Difference (95% CI)	p	GBA1wt	GBA1mut	Difference (95% CI)	p
UPDRS total	4.8	3.1	−1.7 (−0.5 to 2.0)	0.38	18.6	24.5	5.9 (0.3 to 11.7)	0.04
UPDRS I	0.5	0.6	0.1 (−5.4 to 2.0)	0.57	0.0	0.6	0.6 (−0.5 to 1.2)	0.07
UPDRS II	0.9	−0.1	−1.0 (−2.2 to 0.2)	0.10	6.6	7.9	1.3 (−0.5 to 3.0)	0.15
UPDRS III	2.8	2.0	−0.8 (−3.5 to 2.0)	0.59	11.4	15.5	4.1 (0 to 8.3)	0.05
UPDRS IV	0.3	0.3	0.0 (−0.4 to 0.3)	0.84	1.7	2.7	1.0 (0.2 to 1.8)	0.02
Levy A	2.4	1.8	−0.6 (−2.7 to 1.6)	0.60	7.3	10.1	2.8 (−0.5 to 6.0)	0.09
Levy B	0.6	0.4	−0.2 (−0.7 to 0.4)	0.50	2.0	1.4	−0.6 (−2.4 to 1.1)	0.49
LEDD	340	300	−40 (−93 to 13)	0.14	434	462	28 (−104 to 160)	0.67

UPDRS total: the sum of UPDRS subscales I, II and III.

GBA1wt: patients without a *GBA1* polymorphism, mild or severe *GBA1* mutation.

GBA1mut: patients with a *GBA1* polymorphism, mild or severe *GBA1* mutation.

There were no significant differences between GBA1mut and GBA1wt in MoCA score (difference −0.3 points, 95% CI −1.5 to 1.0, p = 0.67) and SCOPA-AUT (0.7 points, 95% CI −1.4 to 2.8, p = 0.52) at five years. At 80 weeks, there was no significant difference between GBA1mut and GBA1wt in prevalence of dyskinesia (respectively 10% and 12%, p = 0.48) and prevalence of motor response fluctuations (respectively 6% and 10%, p = 0.24). At five years, there also was no significant difference between GBA1mut and GBA1wt in prevalence of dyskinesia (respectively 44% and 32%, p = 0.12), though there was a non-significant trend towards a higher prevalence of motor response fluctuations in GBA1mut compared with GBA1wt (respectively 50% and 33%, p = 0.06). At five years, there was also a non-significant trend toward a higher prevalence of hallucinations in GBA1mut compared with GBA1wt (26% vs. 15%, p = 0.07). Details concerning treatment at five years, including medication subtypes, and the number of patients receiving deep brain stimulation or continuous intrajejunal levodopa infusion, are provided in the Supplemental Material.

## Discussion

Our cohort comprised early PD patients who were diagnosed up to two years prior to baseline assessment and who had had insufficient disability at baseline to warrant treatment with antiparkinson medication. We identified a *GBA1* mutation in 13% of the included patients. Our findings indicate a faster progression of total UPDRS score and UPDRS IV score (related to therapy complications) in GBA1mut compared with GBA1wt over the span of five years. However, these disparities were not evident within the initial 80 weeks. We observed a non-significant tendency towards a higher prevalence of motor response fluctuations in GBA1mut versus GBA1wt at five years, a trend not yet noticeable at 80 weeks. At both 80 weeks and five years, we found no between-group differences in prevalence of dyskinesia, cognitive dysfunction, or autonomic dysfunction.

In comparison to data from two incidence cohorts, our study employed more detailed outcome measures—one cohort study utilized the Hoehn and Yahr scale,^3^ while the other used UPDRS II and UPDRS III separately^10^—and had more frequent study visits during the initial 80 weeks. Our findings affirm that around the time of diagnosis (i.e., at start of this study), besides being slightly younger, individuals with GBA1mut are clinically indistinguishable from those with GBA1wt.^3,10^ And that patients with GBA1mut experience more severe symptoms after five years.^1,3–14^ Notably, during the initial 80 weeks after inclusion, there were no discernible differences between the GBA1mut and GBA1wt. A similarity to note is that *LRRK2* risk variant carriers also have a similar motor progression compared to non-carriers during the first four years of the disease, and show more disease progression than non-carriers in the years thereafter.^29^ A possible explanation for this observation in GBA1mut is that disease progression is faster from the beginning but the difference with GBA1wt is yet too small to be detected and/or obscured by use of levodopa early in the disease course, either initiated at baseline or 40 weeks later. Another hypothesis could be that GBA1mut, due to their younger age at diagnosis, possess greater compensatory capacity during the initial stages of the disease.^12^ However, as time progresses, these compensatory mechanisms may become inadequate to counterbalance the progression, leading to the exacerbation of symptoms.^12^ The understanding of the lack of faster disease progression in GBA1mut over the initial 80 weeks contributes to delineating the *GBA1* mutation phenotype and proves valuable in counselling early PD patients with a *GBA1* mutation. Additionally, this information is pertinent for planning and designing clinical trials for potential disease-modifying and glucocerebrosidase-targeted therapies. Research on disease-modifying therapies for PD is challenging due to the large number of participants required and the lengthy follow-up period. Typically, such trials have a follow-up duration of 12 to 24 months.^16^ If a trial were to exclusively enroll patients with a *GBA1* mutation, fewer participants would be needed due to their faster disease progression,^10^ bearing in mind that this acceleration occurs approximately two years after diagnosis, not from the onset of symptoms.

Our study showed a greater deterioration in the UPDRS IV subscale, concerning therapy-related complications, in GBA1mut versus GBA1wt over five years. However, this difference did not stem from a higher prevalence of dyskinesia in GBA1mut compared to GBA1wt at either 80 weeks or five years. There was a trend towards a higher prevalence of motor response fluctuations in GBA1mut versus GBA1wt at five years. Previous studies reporting on motor complications did not present results of the UPDRS IV subscale but focused solely on dyskinesia and motor response fluctuations separately. Therefore, our current results provide novel insights into a broader spectrum of therapy-related complications. Regarding dyskinesia and motor response fluctuations, a similarly sized longitudinal study did not demonstrate earlier onset of dyskinesia or motor response fluctuations in GBA1mut versus GBA1wt.^30^ However, a smaller longitudinal study indicated a higher risk of dyskinesia in GBA1mut versus GBA1wt,^31^ and a slightly larger retrospective study showed earlier onset of motor response fluctuations in GBA1mut.^5^

The absence of differences in cognition at five years between GBA1mut and GBA1wt contrasts with the findings of multiple previous studies, which demonstrated greater and earlier cognitive dysfunction in GBA1mut versus GBA1wt. This discrepancy between our results and previous literature could be attributed to the relative short disease duration of our patients—studies by Cilia et al. indicated a higher incidence of dementia at approximately six years into the disease^9^—and the potential lack of sensitivity of the MoCA. Additionally, our cohort comprised relatively few patients with severe *GBA1* mutations, which is further discussed below.

The current results are based on data from an intervention study, which introduces methodological limitations and advantages. The limitations include: (1) a sample size determined by a power analysis focused on the primary outcome of the LEAP study, and (2) strict inclusion criteria, which may lead to selection bias. However, the study design also offers several advantages: (1) the strict inclusion criteria result in a homogenous sample, (2) the rigorous study protocol included eight visits within the first 80 weeks, providing more detailed data than typically collected in incidence cohorts, where study visits occur only every six months, annually, or at even longer intervals, and (3) detailed documentation of antiparkinson medication at each visit allowed us to adjust our analyses for LEDD on a per-visit basis. The adjustment for LEDD on a per-visit basis not only accounts for the initial group allocation but also captures deviations from the study protocol—approximately 30% of patients in the delayed-start group (initially assigned to placebo) began levodopa treatment during the first 40 weeks, as well as differences in LEDD observed during the follow-up visits at three and five years. One specific inclusion criterium to note is that the study only enrolled patients who had insufficient disability to warrant treatment with antiparkinson medication. This selection criteria might have introduced a bias towards fewer patients with a *GBA1* mutation being included if those with the mutation experience faster disease progression early in the disease course. However, the frequency of patients with a *GBA1* missense mutation in our cohort is similar or even larger compared to other cohorts, including the incidence cohorts.^3,5,6,8–10^ Another limitation is the small number of severe mutations in this cohort which could have resulted in less cognitive decline compared to other studies in which only patients with severe *GBA1* mutations were included. Nevertheless, the frequency of severe and mild mutations in our cohort is consistent with that observed in the two incidence cohorts,^3,10^ thus likely resembles the actual incidence in Europe. Another limitation is the lack of data concerning the prevalence of other mutations that are genetic risk factors for PD in this patient cohort.

The present study showed that PD patients with a *GBA1* mutation, diagnosed up to two years prior, had faster disease progression compared to patients without a *GBA1* mutation over the course of five years. Patients with a *GBA1* mutation also encountered more therapy-related complications at five years. However, these differences were not yet apparent within the initial 80 weeks after inclusion.

## Supplemental Material

sj-docx-1-pkn-10.1177_1877718X251361507 - Supplemental material for Faster disease progression in Parkinson's disease with glucocerebrosidase genotype: But not apparent immediate from diagnosis Supplemental material, sj-docx-1-pkn-10.1177_1877718X251361507 for Faster disease progression in Parkinson's disease with glucocerebrosidase genotype: But not apparent immediate from diagnosis by Henrieke L Frequin, Bart Ferwerda, Constant VM Verschuur, Sven R Suwijn, Joke M Dijk, Rob MA de Bie and in Journal of Parkinson's Disease
