# Supplementary material for: Faster disease progression in Parkinson's disease with glucocerebrosidase genotype: But not apparent immediate from diagnosis
Source: J Parkinsons Dis. 2025 Sep 3;15(7):1208–17. doi: 10.1177/1877718X251361507 (PMC13347520; doi:10.1177/1877718X251361507)
Supplement: sj-docx-1-pkn-10.1177_1877718X251361507 - Supplemental material for Faster disease progression in Parkinson's disease with glucocerebrosidase genotype: But not apparent immediate from diagnosis [file sj-docx-1-pkn-10.1177_1877718X251361507.docx]

**Supplemental Material**

**Faster disease progression in Parkinson’s disease with glucocerebrosidase genotype: But not apparent immediate from diagnosis**

**Supplemental Table 1.** Outcomes mixed-effects regression model, change from baseline to five years, GBA1wt versus patients with a *GBA1* polymorphism and patients with a mild or severe *GBA1* mutation.

|  | GBA1wt (n=342) | 95% CI | GBA1poly (n=44) | 95% CI | p | GBA1m&s (n=8) | 95% CI | | p |
| --- | --- | --- | --- | --- | --- | --- | --- | --- | --- |
| UPDRS total | 18.6 | 16.2 to 21.0 | 24.4 | 15.9 to 32.9 | 0.06 | 25.2 | 8.4 to 42.1 | | 0.37 |
| UPDRS I | 0 | 0 to 0.3 | 0.6 | 0 to 1.6 | 0.09 | 0.6 | 0.1 to 2.4 | | 0.52 |
| UPDRS II | 6.6 | 5.9 to 7.4 | 7.6 | 5.1 to 10.2 | 0.29 | 9.6 | 4.5 to 14.6 | | 0.19 |
| UPDRS III | 11.4 | 9.6 to 13.1 | 15.6 | 9.5 to 21.8 | 0.06 | 14.4 | 2.2 to 26.7 | | 0.57 |
| UPDRS IV | 1.7 | 1.4 to 2.0 | 2.9 | 1.7 to 4.0 | 0.01 | 1.4 | 0 to 3.7 | | 0.77 |
| UPDRS total: the sum of UPDRS subscales I, II and III.  GBA1wt: patients without a *GBA1* polymorphism, mild or severe *GBA1* mutation.  GBA1poly: patients with a *GBA1* polymorphism.  GBA1m&s: patients with a mild or severe *GBA1* mutation. | | | | | | | |  |  |

**Supplemental Table 2.** Treatment at five years.

|  | GBA1wt | GBA1mut |
| --- | --- | --- |
| Total, N | 263 | 43 |
| DBS, N (%) | 3 (1) | 0 |
| Continuous intrajejunal levodopa, N (%) | 2 (1) | 1 (2) |
| Levodopa, N (%) | 258 (98) | 43 (100) |
| Dopamine agonist, N (%) | 47 (18) | 11 (26) |
| COMT-I, N (%) | 16 (6) | 4 (9) |
| MAOB-I, N (%) | 1 (0) | 0 (0) |
| Amantadine, N (%) | 7 (3) | 2 (5) |

This table shows the number of patients who use a certain form of treatment at five years.

GBA1wt: patients without a *GBA1* polymorphism, mild or severe *GBA1* mutation.

GBA1mut: patients with a *GBA1* polymorphism, mild or severe *GBA1* mutation.
